# Gene Expression and Mutational Landscape in a PMEC Patient With Low to Intermediate-High Grade Transition

**DOI:** 10.3389/fonc.2022.820966

**Published:** 2022-03-22

**Authors:** Weijie Dong, Bing Zhu, Yan You, Bing Wang, Dan Fu, Daoxin Wang, He Li, Changyi Li

**Affiliations:** ^1^ Department of Respiratory and Critical Care Medicine, The Second Affiliated Hospital of Chongqing Medical University, Chongqing, China; ^2^ Department of Thoracic Surgery, The Second Affiliated Hospital of Chongqing Medical University, Chongqing, China; ^3^ Department of Pathology, The Second Affiliated Hospital of Chongqing Medical University, Chongqing, China; ^4^ The Medical Department, 3D Medicines Inc., Shanghai, China

**Keywords:** primary pulmonary mucoepidermoid carcinoma, CRTC1-MAML2 and CRTC3-MAML2 fusion, WES-seq, RNA-seq, PDPK1

## Abstract

Primary pulmonary mucoepidermoid carcinoma (PMEC) is a very rare form of lung carcinoma. Due to the low incidence, little is known about its inherent genetic variation characteristics. The uniform treatment for PMEC has not been determined. In this case, we present a 45-year-old male with stage IA PMEC. The surgical specimens contained changes from low- to intermediate-to-high grade. We performed integrative analysis of whole-exome sequencing (WES-seq) and messenger ribonucleic acid sequencing (RNA-seq) to compare the molecular changes in the different lesions. Molecular testing exhibits the specimens harboring CRTC3-MAML2 fusion. The copy number gain of PDPK1 is only present in high-grade regional specimens. We also explored the level of immune infiltration by CIBERSORT. To our knowledge, this is the first report to describe a case of PMEC in the low- to intermediate–high-grade transition with multiomics analysis.

## Introduction

Primary pulmonary mucoepidermoid carcinoma (PMEC) is very rare that it accounts for only 0.1% to 0.2% in lung carcinomas (1). Most pulmonary MECs arise from the submucosal glands of the proximal bronchi. In terms of histological characteristics, MEC is a combination of mucus secreting cells, squamous cells, and intermediate cell types. According to histological features, PMEC is classified into low, intermediate, and high grades (2). This tumor is considered inert and usually carries a better prognosis. Surgical resection is the main treatment for low-grade PMEC, while high-grade PMEC is a more aggressive malignant tumor often accompanied with metastasis. However, high-grade PMEC responds differently to chemotherapy; a deeper understanding of the molecular basis of this cancer is needed.

The important specific genetic aberration in mucoepidermoid carcinoma (MEC) is chromosomal fusion. CRTC1/3-MAML2 fusion has been found to express specificity in the MEC. Recent studies have identified that CRTC1-MAML2 fusion is the main carcinogenic driver of MEC establishment, and p16-CDK4/6-RB activity acts as the cooperative pathway (3). However, the role of CRTC3-MAML2 fusion in the development of MEC, especially in PMEC, is still unclear. Herein, we report a patient with PMEC harboring the CRTC3-MAML2 fusion to analyze the genomic and transcriptional heterogeneity, and PDPK1 copy number variation was identified in the low- to intermediate–high-grade transition by multiomics analysis.

## Case Description

**Figure 1:**
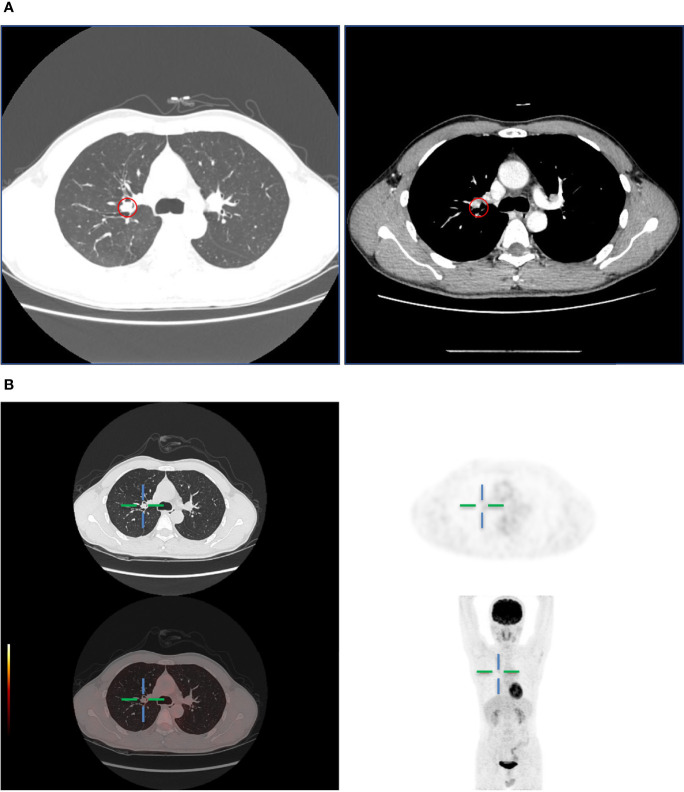
**(A)** Computed tomography showed a lung mass in the upper right lobe. **(B)** PET-CT showed mild metabolically active lung mass.

**Figure 2:**
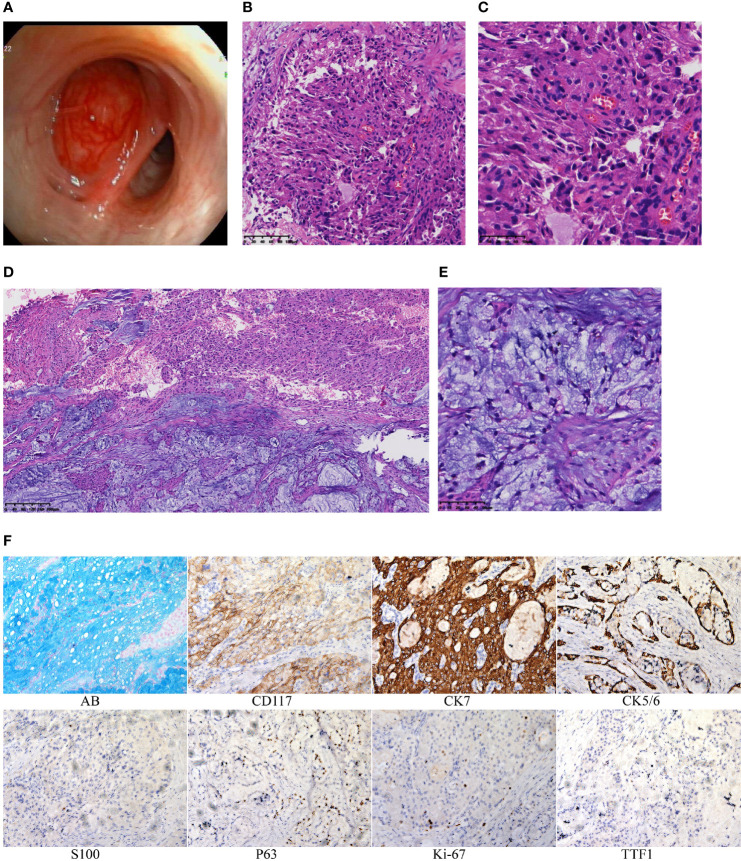
**(A)** The organisms protruding in the bronchus. **(B)** Histologic section showing the oncocytic MEC obtained by transbronchial forceps biopsy (hematoxylin and eosin staining 20×). **(C)** Histologic section showing the oncocytic MEC obtained by transbronchial forceps biopsy (40×). **(D)** Histologic section showing the PMEC obtained by surgery (10×). **(E)** Histologic section showing the PMEC obtained by surgery (40×). **(F)** By immunohistochemistry, tumor cells are positive for AB\CD117\CK7\CK5/6\P63\Ki-67 and negative for TTF1\S100.

A 45-year-old man presented with cough and hemoptysis for more than 10 days. Chest computed tomography demonstrated a 10.4 mm*7.4 mm nodule in the right upper lobe lung (**Figure 1A**), and positron emission tomography CT showed a mild fluorodeoxyglucose uptake (standardized uptake value max 2.1) (**Figure 1B**). A transbronchial forceps biopsy of the lung nodule revealed the malignant cells with a moderate amount of eosinophilic cytoplasm (**Figure 2A**), which was diagnosed as the eosinophilic tumor (**Figures 2B, C**). The patient had undergone right upper lobectomy for stage IA, and the pathological diagnosis was mucoepidermoid carcinoma comprising mucinous cancer cells and oncocytic cancer cells (**Figures 2D, E**; **Supplementary Figure S1**). Postoperative immunohistochemical analysis revealed the stain positive for Alcian Blue (AB)\CD117\CK7\CK5/6\P63\Ki-67 and negative for TTF 1 and S100 (**Figure 2E**).

The presence of mucus-producing cells indicates low-grade mucoepidermoid carcinoma. Eosinophilic MEC suggests intermediate-to-high-grade mucoepidermoid carcinoma (1). Oncocytic mucoepidermoid carcinoma (OMEC) is a variant of MEC that, although higher grade, often behaves indolently (4, 5). Therefore, after surgical complete resection, no further treatment was given to the patient. Up to now, there is no evidence of recurrence or metastasis for 12 months. To determine the inner malignant conversion mechanism of PMEC, we performed integrative analysis of whole-exome (WES) and messenger ribonucleic acid sequencing (RNA-seq) to compare the molecular changes of mucinous cancer cell areas and oncocytic cancer cell regions.

**Figure 3:**
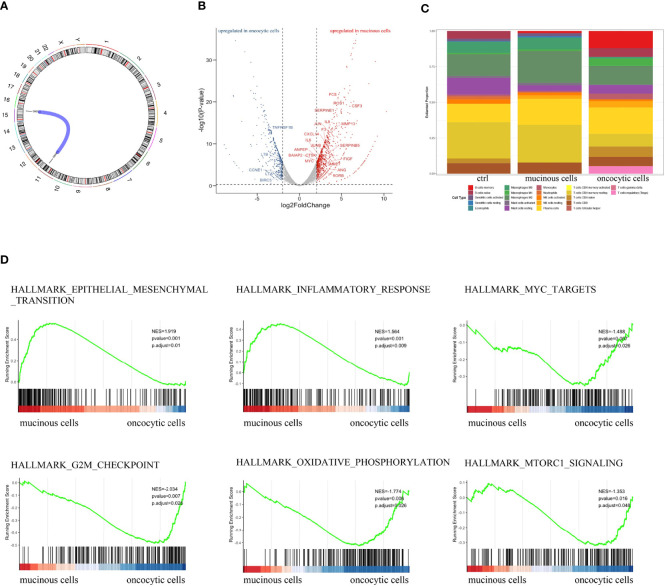
**(A)** RNA-seq showed the fusion gene in both fractions of PMEC. **(B)** Heatmap of cancer-related genes in the two lesions of PMEC. **(C)** The immune microenvironment of the PMEC in the two parts of PMEC. **(D)** The tumor-related gene signatures in the PMEC.

We analyzed differentially expressed genes in the mucinous cancer cells and oncocytic cancer cells. The t(15;11) translocation resulting in CRTC3-MAML2 fusion was identified in both lesions (**Figure 3A**). Among cancer-related genes, genes related to immune response and cell growth were significantly upregulated in the oncocytic cancer cells, whereas genes correlated with cell differentiation, cellular transformation, cell adhesion, and metabolism were upregulated in the mucinous cancer cells (**Figure 3B**). To further explore the immune microenvironment of the PMEC, we analyzed the immune infiltration in the eosinophil and mucinous cancer cells with CIBERSORT (6). The composition of immune cells in different lesions varies greatly. We also observed a trend toward less CD8+ T cell infiltration in the two components (**Figure 3C**). These data indicate that with such immune microenvironment, immunotherapy may be difficult to play a tumoricidal effect to improve the PMEC treatment. We further explored several tumor-related gene signatures (**Figure 3D**). Both the epithelial mesenchymal transition gene signature (p<0.001) and the inflammatory response gene signature (p<0.001) were markedly enriched in the mucinous cancer cells, while oncocytic cancer cells were significantly involved in the positive regulation of Myc targets (p<0.001), G2M checkpoint (p<0.001), and oxidative phosphorylation (p<0.001) processes. These data revealed that RNA expression pattern was related to tumor differentiation.

**Figure 4:**
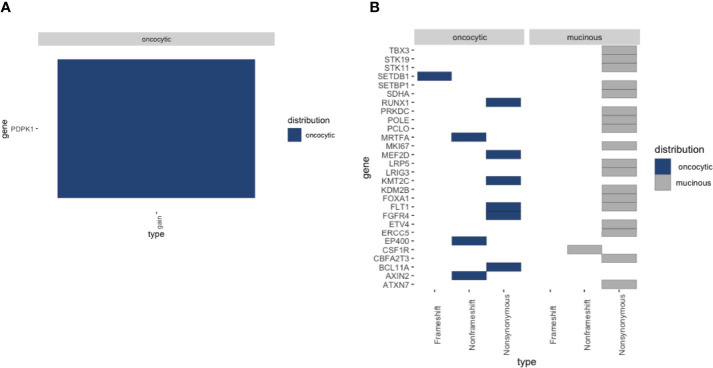
**(A)** Heatmap of the copy number variation (CNV) mutations detected in oncocytic MEC and mucocyte MEC. **(B)** Heatmap of the single nucleotide variation (SNV) detected in oncocytic MEC and mucocyte MEC.

To further explore the genomic profiles, WES-seq was applied to the two lesions of PMEC. We explored the nonsilent somatic mutations of tumor-related genes across the two fractions and found that the mutations in different regions were mainly involved in the Wnt signaling pathway, DNA-binding transcription factor activity, and protein kinase activity (**Figure 4B**). Next we checked the copy number level of tumor-related genes in the PMEC. The copy number gain of PDPK1 only showed in the oncocytic cancer cell specimens (**Figure 4A**). To uncover the subclones present in the PMEC, we analyzed the oncocytic part and the mucinous lesion (7). Two similar groups of mutations existed in the two samples. The heterogeneity observed in the clusters may be due to the low variable percentages of genomic alteration in the lesions.

## Discussion

Primary pulmonary MEC is a relatively infrequent type of lung cancer. Low-grade pulmonary MECs are more indolent. Patients with PMEC stage I–II, as described in this case, usually have optimistic prognosis after surgical resection; the 5-year OS rates were more than 80.0% (8). In contrast, high-grade pulmonary MECs were factually actually more prone to invasive growth and metastasis. Since fewer cases of high-grade pulmonary MEC have been reported, there is no consensus on the treatment of this group. The molecular underpinning of this cancer needs to be explored.

A histopathological examination was performed to this patient diagnosed with PMEC. There is a low- to high-grade transformation from the middle to the outer area. RNA-seq analysis revealed that CRTC3-MAML2 fusion was identified in both lesions. There are three genes, CRTC1, CRTC2, and CRTC3, in the CRTC family. CRTC 1/3-MAML2 fusion is often highly specific in MEC and can be used as a diagnostic marker; the expression of the two fusion genes is mutually exclusive. The prognosis of fusion-positive patients is always better than that of fusion-negative patients (9). In addition, compared with low-grade PMEC tumors, including Myc targets, G2M checkpoint and oxidative phosphorylation processes were significantly upregulated in high-grade tumors. We also observed a trend toward less CD8+ T-cell infiltration in the two components, indicating that immunotherapy may not be effective.

In the whole-exome sequencing study, no TP53 mutation was detected in the PMEC, which is consistent with previous reports (10). The most common mutation in MEC is TP53. Thus, TP53 variation is different between MEC in lung and MEC in other parts. Copy-number analysis revealed that PDPK1 amplification was the only potentially tumor-related gene in the oncocytic cancer cell specimens. PDPK1 is related to signal pathways that are frequently changed in cancer, such as PI3K/Akt, Ras/MAPK, and Myc, as well as with poor prognosis (11, 12). This is consistent with the activation of tumor-related gene signatures in high-grade PMEC regional specimens. Therefore, it can be speculated that PDPK1 may be a potentially significant gene in the low- to intermediate–high-grade transition.

In summary, these preliminary findings provided a detailed view of the pathogenesis of PMEC, including the mutation spectrum, and immune microenvironment profiles of multifocal PMEC by multiomics analysis. The CRTC3–MAML2 fusion might drive oncogenesis, and PDK1 may play a role in the evolution of PMEC. This case report could expand the genomic landscape of CRTC3–MAML2 fusions and reveal the development of evolutionary mechanisms. Further exploration of the molecular characteristics of PMECs may identify new targets.

## Data Availability Statement

The original contributions presented in the study are publicly available. This data can be found here: NGDC, BioProject, PRJCA007683.

## Author Contributions

WD: Writing—original draft. BZ and BW: Data curation. DF: Software and validation. DW: Supervision. YY: Investigation. HL and CL: Writing—review and editing. All authors contributed to the article and approved the submitted version.

## Funding

This work was supported by the Chongqing Natural Science Foundation of China [cstc2020jcyj-msxmX0472].

## Conflict of Interest

Author DF was employed by The Medical Department, 3D Medicines Inc.

The remaining authors declare that the research was conducted in the absence of any commercial or financial relationships that could be construed as a potential conflict of interest.

## Publisher’s Note

All claims expressed in this article are solely those of the authors and do not necessarily represent those of their affiliated organizations, or those of the publisher, the editors and the reviewers. Any product that may be evaluated in this article, or claim that may be made by its manufacturer, is not guaranteed or endorsed by the publisher.

## Supplementary Material

Supplementary Figure 1 Histologic section showing eosinophilic and necrotic areas (*) obtained by surgery (10×).
