# Supplementary figures and images for: Gene Expression and Mutational Landscape in a PMEC Patient With Low to Intermediate-High Grade Transition
Source: Front Oncol. 2022 Mar 22;12:820966. doi: 10.3389/fonc.2022.820966 (PMC8980482; doi:10.3389/fonc.2022.820966)

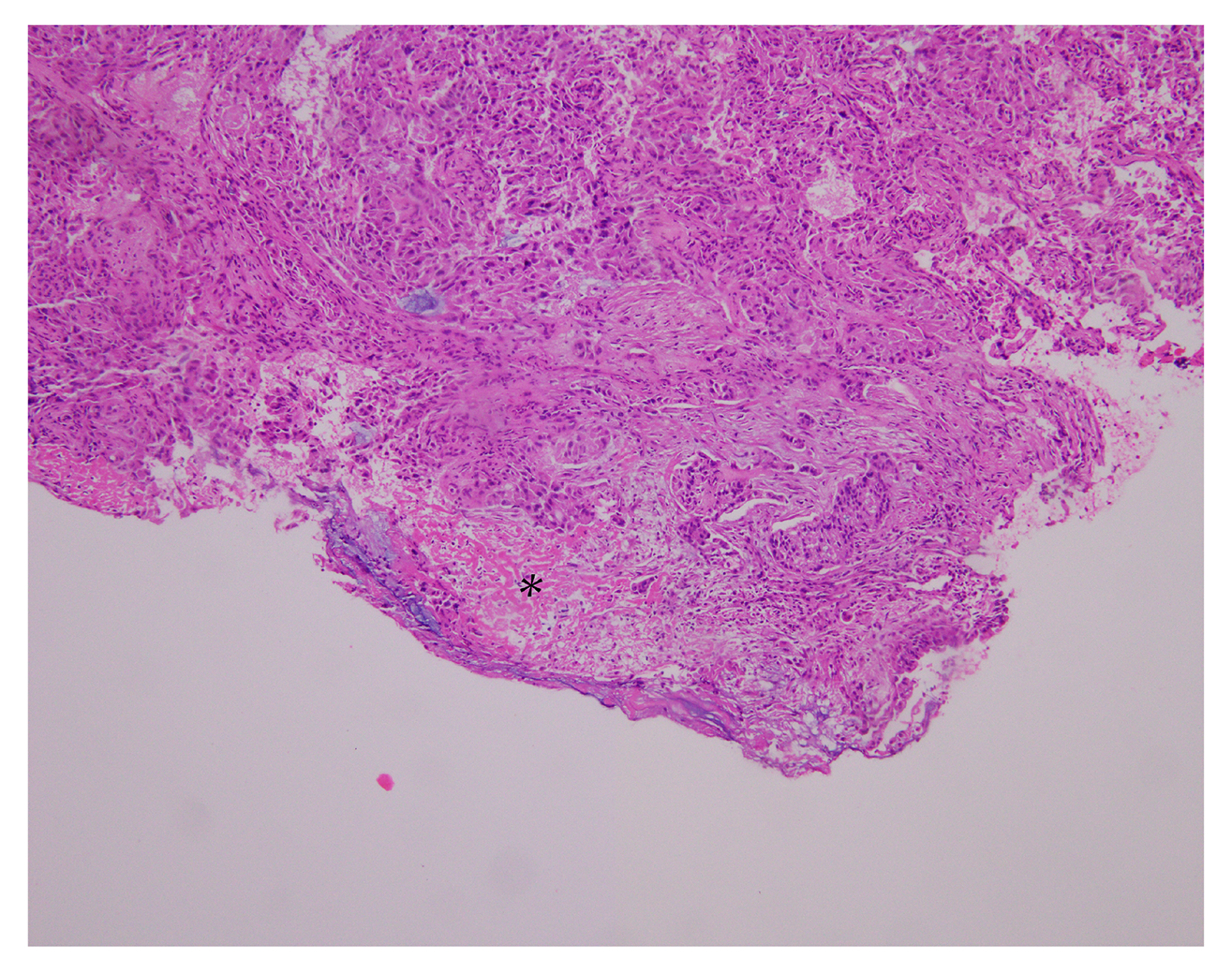

Supplement: Supplementary Figure 1 — Histologic section showing eosinophilic and necrotic areas (*) obtained by surgery (10×). [file Image_1.tif]
